# Prevalence and risk factors of loneliness among older adults in China: a nationally representative study

**DOI:** 10.1186/s12877-026-07531-6

**Published:** 2026-04-28

**Authors:** Bin Yu, Sixin Liu, Siyuan Zhang, Julianne Holt-Lunstad, Hui Zhu

**Affiliations:** 1 School of Education, Tianjin University, Tianjin, China; 2 Institute of Applied Psychology, Tianjin University, Tianjin, China; 3 Department of Psychology, Brigham Young University, Provo, USA; 4 School of Ethnology and Sociology, Minzu University of China, Beijing, China; 5 School of Sociology, Nankai University, Jinnan Campus, No.38 Tongyan Road, Haihe Education Park, Tianjin, China

**Keywords:** Loneliness, Prevalence, Risk factors, Older adults, China

## Abstract

**Background:**

Loneliness is increasingly recognized as a significant public health concern, yet limited research has examined its prevalence and risk factors among older adults in China.

**Methods:**

This study investigated the prevalence and risk factors of loneliness among older adults in China using data from the 2018 China Longitudinal Aging Social Survey (CLASS), a nationally representative study. The sample included 9,951 participants aged 60 and older (mean age = 71.3 years, SD = 7.3; 49.94% female). Loneliness was measured using the Chinese version of the three-item UCLA Loneliness Scale, which was validated as a reliable and effective tool for assessing loneliness in this population.

**Results:**

Overall, 19.6% of older Chinese adults reported experiencing loneliness. Logistic regression models identified demographic factors (older age, living in rural areas, lower income, and lower education), health factors (depressive symptoms and functional limitations), and social factors (living alone, reduced social support and social participation) as being associated with loneliness. Among these, higher levels of depressive symptoms (OR 1.29, 95% CI 1.26–1.32), living alone (OR 1.37, 95% CI 1.14–1.65), and reduced social participation (OR 1.51, 95% CI 1.35–1.71) emerged as the strongest predictors of loneliness.

**Conclusions:**

The findings highlight the high prevalence of loneliness among older Chinese adults and underscore the need for targeted interventions, especially for individuals with depressive symptoms, those living alone, or those with limited social engagement.

## Introduction

Loneliness occurs when there is a discrepancy between an individual’s actual and desired social relationships [1, 2]. An increasing body of research links loneliness to a range of adverse health outcomes, including mental health conditions such as depression, anxiety, and sleeping disturbances [3, 4], as well as physical problems like cardiovascular diseases, dementia, endocrine and immune disorders [5, 6]. A 2015 meta-analysis revealed that loneliness increases the risk of mortality by 26%, a risk comparable to established factors like smoking, physical inactivity, and obesity [7]. Loneliness has been increasingly recognized as a critical public health issue in many Western countries. For instance, the British government appointed a Minister for Loneliness in 2018. Similarly, the U.S. Surgeon General has referred to loneliness as an epidemic with serious health consequences [8].

Although loneliness affects people of all ages, it is particularly prevalent among older adults. Estimates of loneliness among older people vary significantly across nations. While there are no global assessments of loneliness, the WHO estimates that 20% to 34% of older people experience loneliness [9]. A recent meta-analysis, which included data from 106 countries and 24 studies, suggests that older adults (aged 60 years and above) tend to experience higher loneliness rates than younger populations [10]. However, much of the research included in this analysis has been conducted in Western countries, and only a few nationally representative studies have been carried out in less developed regions.

China is undergoing profound demographic and societal transformations, including rapid population aging, internal migration, declining fertility rates, and shrinking household size [11]. These changes have contributed to a growing number of older adults living alone. The implications of living alone in Chinese society may differ from those in Western contexts due to traditional cultural values. For instance, as a core value in Chinese culture, filial piety remains important to many older adults, reinforcing the belief that family is the primary source of social support. As a result, older adults who live alone and lack adequate family support may face increased vulnerability to loneliness in China [12]. Given the increasing trend of older adults living independently, it is crucial to examine both the prevalence of loneliness and the factors contributing to it in China’s evolving social landscape.

While several studies have examined the prevalence of loneliness in the Chinese population, many are limited by methodology constraints. First, the measurement tools used in these studies are often inadequate. Many rely on single-item questions, such as ‘How often do you feel lonely?’ [13, 14]. These direct measures are susceptible to reporting biases [15], particularly in China, where loneliness carries cultural stigma, and individuals may be reluctant to disclose negative feelings in surveys. In contrast, the three-item UCLA Loneliness Scale, adapted from the original 20-item UCLA Loneliness [16], avoids the use of stigmatizing terms such as “lonely” and instead assesses loneliness indirectly through related experiences. This scale was specially designed for large-scale surveys [17] and has been widely used in major Western aging cohorts such as the Health and Retirement Study (HRS) in the United States and the English Longitudinal Study of Ageing (ELSA) [18, 19]. To the best of our knowledge, the China Longitudinal Aging Social Survey (CLASS) is among the few cohort studies in China to incorporate the three-item UCLA Loneliness Scale [20, 21], using a relatively large and nationally representative sample. While several studies have examined the association between loneliness and health outcomes using CLASS data, the psychometric properties of the scale have yet to be fully evaluated, and no studies have explored the prevalence of loneliness using this dataset.

Second, existing studies suffer from small, unrepresentative samples. Most studies relied on convenience samples or were confined to specific regions, limiting the generalizability of their findings. For example, a significant proportion of research has focused on “empty-nest” older populations [22–24]. Empty nesters are aging individuals who are either single or living solely with their spouse, as their children live away from home [22]. However, older adults without children are excluded from these studies, potentially failing to capture the broader, national experience of loneliness among older adults in China. Additionally, many of these studies were conducted over a decade ago [11, 13], failing to account for the rapid societal and demographic transformations that have occurred in recent years.

Third, although previous studies have explored the risk factors for loneliness among older Chinese adults, most of them are based on small sample sizes or less valid measures of loneliness [13, 25]. While meta-analyses and systematic reviews in Western populations consistently identify key risk factors for loneliness — such as low socioeconomic status, widowhood, poor physical health, disability, and poor-quality social relationships [26, 27] — these factors may not fully apply in the Chinese context. Cultural differences play a significant role in shaping the experience of loneliness [27]. In China, the emphasis on interdependence, family ties, and mutual support within social networks may be more influential in determining loneliness levels than in Western countries [13].

Therefore, an updated study that reflects the current realities in China is crucial for providing more accurate insights into the prevalence and risk factors of loneliness among Chinese older adults. Using data from the China Longitudinal Aging Social Survey (CLASS), a large, nationally representative sample of older adults, we first evaluated the psychometric properties of the Chinese version of the three-item UCLA Loneliness Scale. Following this, we examined the prevalence and risk factors of loneliness among older adults in China.

## Materials and methods

### Participants

This study used data from the 2018 China Longitudinal Aging Social Survey (CLASS), conducted by Renmin University of China. CLASS is a nationally representative study that employed a stratified multi-stage probability sampling design. The survey covers 28 provinces in China, comprising 462 villages, neighborhoods, and communities nationwide. Data were collected through face-to-face interviews conducted by trained interviewers, with field supervision, telephone verification, and remote data checks implemented to ensure data quality. The CLASS project first conducted a baseline survey in 2014 and follow-up surveys in 2016, 2018, 2020 and 2023. We used the 2018 wave because it is the most recent wave that includes the three-item UCLA Loneliness Scale. In 2018, CLASS surveyed 11,419 individuals. After excluding one respondent under 60 years old and 1,467 respondents without valid data for the loneliness variable, the final sample consisted of 9,951 respondents (mean = 71.3, SD = 7.3).

## Measures

### Loneliness

Loneliness was assessed using the Chinese version of the three-item UCLA Loneliness Scale validated for large-scale surveys [17]. The scale measured how frequently participants experienced feelings such as “lack of companionship,” “being left out,” and “isolated from others,” thereby evaluating loneliness in terms of interactions with intimate others, social others, and the broader environment. Responses were coded as 1 (hardly ever), 2 (sometimes), and 3 (often). The scores for each participant were summed (range = 3–9), with higher scores indicating greater levels of loneliness. To ensure comparability with previous studies in Western countries using the same scale, participants who responded ‘often’ to any of the three items were categorized as “lonely” [28–30]. This approach is based on the premise that each item reflects a distinct manifestation of loneliness rather than additive components of loneliness [30].

### Risk factors

Demographic variables included age, gender, education, living area, and monthly household income (in Chinese yuan). Age was divided into two groups (0 = less than 70 years, 1 = 70 years and above). Education, originally comprising seven levels (illiterate, literacy class, primary school, middle school, high school/vocational school, college, university undergraduate, and above), was recoded into two categories (0 = primary school or above, 1 = below primary school). The living area variable was categorized as 0 for urban and 1 for rural. Monthly household income (yuan) was treated as a continuous variable and log-transformed to account for skewness.

Health variables included self-reported health, functional limitations, and depressive symptoms. Self-reported physical health was measured using a single item, “How would you rate your health?”, with responses ranging from 1 (poor) to 5 (excellent). Functional limitations were evaluated across six activities of daily living (ADLs): eating, bathing, moving, toileting, dressing, and cleaning. Participants with two or more limitations (coded as 1) were compared to those with fewer than two limitations (coded as 0) [18]. Depressive symptoms were assessed using the nine-item Center for Epidemiologic Studies Depression Scale (CES-D), a well-established tool for identifying depressive symptoms in community samples [31]. Respondents answered each question with ‘never,’ ‘sometimes’ or ‘often’, scored as 0, 1, and 2, respectively. The nine items included feeling happy, lonely, upset, enjoying life, having a poor appetite, having sleep difficulties, feeling useless, having nothing to do, and experiencing pleasure during the week before the survey. The lonely item was excluded from the total score, resulting in a final depression score ranging from 0 to 16.

Social characteristics variables included marital status, living arrangement, social support from family and friends, and social participation. Marital status was categorized as currently married/cohabiting (coded as 0) and all other statuses (widowed, divorced, and never married) combined and coded as 1. Living arrangements were assessed by asking participants how many people lived in their households besides themselves, and the number was coded as 1 if the respondents lived alone and 0 otherwise. Social support was measured using the Lubben Social Network Scale (LSNS-6), a six-item questionnaire [32]. Three questions focus on family members/relatives, asking about the frequency of contact, the number of family members/relatives the respondent could rely on for help, and the number they could confide in about personal matters. Responses ranged from 0 (none) to 5 (nine or more), with scores summed to create a family support score (range = 0‒15), where higher scores indicated stronger family support. The same three questions were asked about friends. Social participation was evaluated based on involvement in social activities, including religious events, attending school or training classes for seniors, playing games like mahjong, cards, or chess, helping care for older adults or children (e.g., assisting with shopping or daily care), accompanying others for conversation, volunteering in activities requiring professional skills (e.g., free medical consultations or cultural promotion), and participation in square dancing. Participation was coded as 0 for those who engaged in any of these activities more than once per month and 1 otherwise.

### Statistical analysis

Cronbach’s alpha was calculated to estimate the internal consistency of the scale, alongside item-test correlations and item-rest correlations. To assess the convergent and discriminant validity of the scale, bivariate correlation analyses were conducted using depressive symptoms as a related variable [17, 20].

A graded response model (GRM), an item response theory (IRT) model designed for ordinal response categories, was applied to the three loneliness items [18]. The GRM models the probability of endorsing each response category as a function of an individual’s underlying loneliness level using ordinal logistic regression. Conceptually, it extends the two-parameter logistic (2PL) model, which is commonly used for binary items, to items with ordered response options. In this model, latent loneliness scores were assumed to follow a standard normal distribution (mean = 0, variance = 1). For each item, estimates of discrimination (the slope of the ordinal logistic regression) and difficulty (threshold parameters) were reported.

Sample characteristics concerning demographic, health and social variables were examined. Group differences between lonely and non-lonely individuals were assessed using Chi-square tests for categorical variables and independent samples *t*-tests for continuous variables. Multivariable logistic regression models were used to analyze the associations between risk factors and loneliness, with results presented as odds ratios (ORs) and 95% confidence intervals (CIs). Risk factors were added stepwise. In Model 1, demographic variables including gender, age, household income, living area, and education were included. In Model 2, the health variables (self-reported health, ADLs, and depressive symptoms) were added. Finally, social characteristics (marital status, living alone, family and friends support, and social participation) were incorporated into Model 3. Multicollinearity among predictors was assessed using variance inflation factors (VIF). All VIF values were below 5, indicating no evidence of problematic multicollinearity. All analyses were performed using Stata version 18.0, with psychometric analyses conducted via the *irt* and *difmh* commands. A significance level of *α* = 0.05 was applied.

## Results

### Psychometric properties of the Chinese version of the three-item UCLA Loneliness Scale

Cronbach’s alpha for the three loneliness items was 0.77, which indicates good internal consistency and is consistent with previous studies conducted in Western countries (range: 0.72–0.83 ) [17, 18]. Item-test correlations (the correlation between each item and the total scale) were all above 0.8 (0.82–0.84), while item-rest correlations (the correlation between each item and the score excluding that item) were all above 0.55 (0.55–0.89), with the lowest value (0.55) observed for the item “lack companionship”.

Higher scores on the loneliness scale were significantly associated with greater depressive symptoms, as measured by the 8-item CES-D (*r* = 0.39, *p* < 0.001). Regarding convergent validity, the correlation between the self-reported loneliness item from the CES-D and the three-item UCLA Loneliness Scale (*r* = 0.40, *p* < 0.001) was stronger than the correlations between any other CES-D components and loneliness (*r* = 0.07–0.36, *p* < 0.001). Table 1 shows the results of the GRM analysis. Discrimination was higher for the item “feel left out” (3.61), indicating that it is the most sensitive in detecting differences in loneliness levels. “Lack of companionship” had the lowest discrimination value (1.88), which is still a moderately sensitive item. “Lack of companionship” also had the lowest difficulty (0.05 for ≥ 2 and 1.55 for 3), suggesting that even individuals with relatively low levels of loneliness are likely to choose higher response categories for this item.

**Table 1:** The graded response model fits to the Chinese version of the three-item UCLA Loneliness Scale

Type of loneliness	Response	*N*	%	Discrimination ^a^	Difficulty (≥ 2, = 3) ^b^
Lack of companionship	Hardly ever	5,060	50.85	1.88	0.05, 1.55
	Sometimes	3,620	36.38		
	Often	1,271	12.77		
Being left out	Hardly ever	5,950	59.79	3.61	0.31, 1.60
	Sometimes	3,277	32.93		
	Often	724	7.28		
Isolated from others	Hardly ever	6,426	64.58	3.00	0.47, 1.81
	Sometimes	2,955	29.70		
	Often	570	5.73		

^a^ The estimated log-odds change for responses exceeding a specific threshold corresponds to a one standard deviation increase in latent loneliness

^b^ The estimated latent loneliness value at which an individual has an equal (50%) likelihood of selecting a given category or a higher one (2 = “sometimes,” 3 = “often”)

### Sample characteristics

The characteristics of the sample are summarized in Table 2. The mean age was 71.31 years (SD = 7.31), with 49.94% of the sample being female, and most participants (58.03%) resided in urban areas. Frequent loneliness was more prevalent among individuals who were older, lived in rural areas, had lower education attainment and household income, reported better health, had higher levels of depressive symptoms, were unmarried, lived alone, had less family and friends support, and participated less frequently in social activities (Table 2). Compared with those who were excluded from the original sample (*n* = 1,467), participants included in the study were more likely to be younger (49.24% vs. 43.42%; *p* < 0.001) and to live in urban area (58.03% vs. 55.28%; *p* = 0.005). They were less likely to report ADLs (19.47% vs. 22.49%; *p* = 0.007) and had better self-report health (3.34 vs. 3.14; *p* < 0.001), but higher depressive symptoms (6.08 vs. 4.57; *p* < 0.001). In terms of social variables, only family support was lower in the included group (7.29 vs. 7.71; *p* < 0.001).

**Table 2:** Characteristics of participants according to categories of loneliness

Characteristics	All	Not lonely	Lonely	*p*
N	9951	8004	1947	
Demographic variables				
Gender (females, %)	49.94	49.85	50.33	0.702
Age (< 70 years, %)	49.24	50.16	45.45	< 0.001
Log household income (yuan)	9.80 (1.62)	9.84 (1.60)	9.63 (1.68)	< 0.001
Living area (urban, %)	58.03	58.77	55.01	0.003
Education (≥ primary school, %)	33.72	35.21	27.58	< 0.001
Health variables				
Self-reported health	3.34 (0.89)	3.35 (0.89)	3.24 (0.89)	< 0.001
ADLs (< 2 limitations, %)	95.38	95.36	95.43	0.904
Depressive symptoms	6.08 (2.79)	5.74 (2.74)	7.50 (2.54)	< 0.001
Social variables				
Marital status (married or living with partner, %)	69.49	70.64	64.77	< 0.001
Living alone (no, %)	87.84	89.03	82.95	< 0.001
Family support	7.29 (2.83)	7.46 (2.77)	6.62 (2.94)	< 0.001
Friends support	6.34 (3.22)	6.45 (3.19)	5.87 (3.31)	< 0.001
Social participation in the last year (yes, %)	48.11	50.20	39.50	< 0.001

Data presented as percentages or mean (SD) values

### Prevalence of loneliness and its risk factors

The mean score (SD) of the loneliness scale was 4.51 (1.59). Participants who responded ‘often’ to any of the three items were classified as experiencing loneliness, resulting in a prevalence rate of 19.6% among older Chinese adults. Regarding specific symptoms, 12.8% of the participants reported a “lack of companionship,” 7.3% felt “left out,” and 5.7% felt “isolated from others” (Table 1).

Table 3 presents results from a series of logistics models incorporating an additional set of covariates. In Model 1, which included demographic variables, older age (OR 1.23, 95% CI 1.10–1.37), lower incomes (OR 0.95, 95% CI 0.92–0.99), and lower educational attainment (OR 1.24, 95% CI 1.10–1.41) were all significantly associated with a higher likelihood of experiencing loneliness. In Model 2, the association between these demographic factors and loneliness largely diminished after adjusting for health characteristics. Depressive symptoms were strongly associated with increased loneliness (OR 1.0, 95% CI 1.27–1.33) while having more ADLs was linked to lower loneliness (OR 0.64, 95% CI 0.48–0.86). These health-related variables remained significant even after adjusting for social characteristics in Model 3. Most social characteristics, except for marital status (OR 0.96, 95% CI 0.83–1.12), were significantly associated with loneliness. The strongest predictors of loneliness were living alone (OR 1.37, 95% CI 1.14–1.65) and lack of participation in social activities (OR 1.51, 95% CI 1.35–1.71). Additionally, greater social support from family (OR 0.90, 95% CI 0.88–0.93) was associated with a reduction in loneliness, while support from friends was unexpectedly associated with a slight increase in loneliness (OR 1.04, 95% CI 1.01–1.06).

**Table 3:** Multiple-adjusted ORs for risk factors associated with loneliness

	Model 1			Model 2			Model 3		
	OR	95% CI	*p*	OR	95% CI	*p*	OR	95% CI	*p*
Demographic variables									
Males (vs. females)	0.99	0.89–1.01	0.851	1.02	0.91–1.14	0.739	1.02	0.91–1.15	0.757
Age (vs. < 70 years)	1.23	1.10–1.37	< 0.001	1.09	0.98–1.22	0.045	1.08	0.96–1.22	0.192
Log household income (yuan)	0.95	0.92–0.99	0.008	1.00	0.96–1.03	0.962	1.03	0.99–1.07	0.180
Living area (vs. rural)	0.87	0.77–0.99	0.031	0.94	0.82–1.06	0.271	0.96	0.84–1.09	0.537
Education (vs. ≥ primary school)	1.24	1.10–1.41	0.001	1.13	0.99–1.28	0.067	1.08	0.95–1.23	0.255
Health variables									
Self-reported health (1–5)				0.99	0.93–1.07	0.882	0.99	0.93–1.06	0.844
ADLs (vs. < 2 limitations)				0.64	0.48–0.86	0.003	0.67	0.50–0.91	0.009
Depressive symptoms (0–16)				1.30	1.27–1.33	< 0.001	1.29	1.26–1.32	< 0.001
Social variables									
Marital status (vs. married)							0.96	0.83–1.12	0.603
Living alone (vs. no)							1.37	1.14–1.65	0.001
Family support (0–15)							0.90	0.88–0.93	< 0.001
Friends support (0–15)							1.04	1.01–1.06	0.004
Social participation (vs. yes)							1.51	1.35–1.71	< 0.001

## Discussion

Loneliness has been recognized as a significant public health issue globally. While it has been extensively studied in Western countries, limited research has focused on its prevalence and risk factors among older adults in China. This population-based study provides detailed data on the experience of loneliness among a nationally representative sample of Chinese older adults. Our first key finding is that loneliness among this population can be reliably measured using the Chinese version of the three-item UCLA Loneliness Scale. Comparable to previous studies conducted in Western populations [17, 18], the scale demonstrated satisfactory reliability, discriminant and convergent validity.

Our second key findings suggest that approximately 20% of older adults in China reported experiencing loneliness, with ‘lack of companionship’ being the most frequently reported symptom (12.7%). In contrast, a previous study of older adults in Mainland China reported a loneliness prevalence of 29.6% in 2000, although this was measured using a single question, which may not fully capture the complexity of loneliness [13]. A recent meta-analysis of studies using the three-item UCLA Loneliness Scale in Western populations (from the US, UK, Finland, and Spain) reported loneliness prevalence rates ranging from 5.9% to 19.3% [10]. However, a study that used the same method of categorizing loneliness as our study (based on at least one ‘often’ response) reported prevalence rates of 11.6% in England and 16.1% in the United States [28], which are relatively lower than our findings. It is also worth noting that participants included in our analysis were healthier and had higher levels of family support than those excluded, which may have led to an underestimation of loneliness prevalence.

The prevalence of loneliness among Chinese older adults may be influenced by cultural and societal factors that overlap with those in Western countries while also exhibiting unique characteristics. In China, traditional family structures, such as multigenerational households, have historically provided older adults with consistent companionship and support, helping to mitigate loneliness. However, rapid urbanization and increased migration of younger generations to urban centers have weakened these structures, leaving many older adults isolated in ‘empty-nest’ households [11, 33]. This trend mirrors patterns observed in Western countries, where geographic mobility and nuclear family living arrangements often lead to higher rates of older adults living alone [34]. Moreover, cultural values in China, such as filial piety, may have traditionally served as a buffer against loneliness [35]. However, with modernization and urban lifestyles, these values are evolving, which may further contribute to the rising prevalence of loneliness among Chinese older adults.

We further investigated the risk factors for loneliness among Chinese older adults. Our findings largely align with prior research on the association between loneliness and demographic characteristics [18, 36, 37]. Consistent with earlier studies in both Western [18] and Chinese populations [38], we found that older age was associated with a higher likelihood of reporting loneliness. As people age, the loss of significant others, combined with declines in physical and mental health, often limits their ability to engage in social activities, which can intensify feelings of loneliness. Additionally, individuals living in rural areas were more likely to experience loneliness than those in urban areas, mirroring findings from previous studies on Chinese populations [13, 39]. Our study did not reveal significant gender differences in loneliness, which aligns with some prior research [40] but contrasts with others [41]. A recent meta-analysis found that while males were slightly lonelier than females in younger age groups, these small gender differences disappeared in middle and older adulthood [42]. Interestingly, we found significant gender differences (t = 2.53, *p* = 0.006) for the loneliness item in the CES-D scale, with women (M = 0.58, SD = 0.67) reporting higher levels of loneliness than men (M = 0.55, SD = 0.66). This may suggest that men are less likely than women to report feelings of loneliness directly.

When health characteristics such as self-rated health, ADLs, and depressive symptoms were adjusted, they accounted for a substantial portion of the demographic effects, suggesting that these factors may mediate the relationship between demographic variables and loneliness. For instance, individuals with higher education and income tend to have better health outcomes, which may reduce their risk of loneliness [43]. While participants with better self-rated health were less likely to report loneliness, this relationship was insignificant once other covariates were controlled. In contrast, depressive symptoms emerged as a strong predictor of loneliness, consistent with findings from previous studies [17, 40]. Surprisingly, ADLs were associated with a lower risk of loneliness, which contrasts with Western studies that have found either a positive relationship [18] or no significant association [40] between ADLs and loneliness. These differences in findings may reflect cultural variations in caregiving practices. Unlike the more individualistic caregiving structures typical of Western countries, older adults in China with limited ADLs often receive substantial support from family members [44], which may enhance social interaction and help reduce feelings of loneliness. Notably, further adjustment for social characteristics did not alter the relationship between depressive symptoms and ADLs with loneliness, indicating that these two factors independently influence loneliness.

All social characteristics were significantly associated with loneliness in univariate analyses, although the association with marriage became insignificant when other covariates were considered. While marriage is generally viewed as protective against loneliness [25], the quality of the marital relationship may play a more critical role. Some studies have found that marriage does not offer a protective effect if the spouse is not considered a confidant [36]. Both living alone and reduced social participation have consistently been identified as risk factors for loneliness [45], and these factors also emerged as strong predictors of loneliness in our study. In Chinese culture, family ties and social networks have traditionally provided emotional and practical support for older adults, but rapid urbanization and the rise of “empty-nest” households have weakened these structures. This shift has intensified the impact of living alone, making it a significant predictor of loneliness. Additionally, the cultural stigma surrounding loneliness may discourage older adults from admitting their feelings or seeking help, further isolating them. As modernization reduces opportunities for traditional social participation, older adults face increasing challenges in maintaining meaningful social connections. Interestingly, support from friends was associated with a slight increase in loneliness. One possible explanation is reverse causality, whereby individuals experiencing greater loneliness may actively seek emotional support from friends. In this case, higher levels of reported support from friends may reflect a response to loneliness rather than a protective factor against it. Another explanation may relate to cultural context. In China, family relationships traditionally play a central role in providing emotional and instrumental support for older adults [32, 46]; therefore, support from friends may not fully compensate for deficits in family support.

These findings highlight the need for targeted interventions to reduce loneliness among Chinese older adults. Based on our results, several strategies may be particularly relevant. First, community-based programs that promote social participation, such as group exercise activities, square dancing, and educational classes for older adults, may help strengthen social engagement. Second, older adults living alone may benefit from targeted outreach programs, including regular community visits or volunteer companionship services. Third, integrating loneliness screening and mental health support into primary care and community health services may help identify and support older adults experiencing both loneliness and depressive symptoms.

This study has several limitations. First, it relies on cross-sectional data, which captures loneliness at a single time point. Although the data collection occurred relatively recently, this approach does not allow for examining how loneliness evolves, particularly in the context of China’s rapidly changing societal, economic, and familial structures. Future studies with longitudinal data would offer a more comprehensive understanding of loneliness trajectories, enabling researchers to better understand how these ongoing changes influence patterns of loneliness. Such insights are essential for designing targeted interventions aimed at mitigating loneliness among older adults in China as they navigate these broader societal transformations. Second, our study focused solely on individual-level risk factors for loneliness. Future research should also consider environmental and neighborhood characteristics that may impact loneliness differently across subgroups and birth cohorts. By examining a wider range of risk factors, researchers can identify populations at greatest risk for chronic or increasing loneliness. Third, this study was representative of older adults in Mainland China; however, its findings should not be generalized to other Chinese populations in other areas. For instance, Chinese populations in Hong Kong, Taiwan, and Singapore may experience loneliness differently due to variations in familial structures, levels of urbanization, and access to social services. Future studies are necessary to explore loneliness among diverse global Chinese populations.

## Conclusions

In sum, this study demonstrates that the three-item UCLA Loneliness Scale is a reliable and valid measure of loneliness among Chinese older adults. Using this scale and a large representative sample, we found that loneliness is prevalent in this population. Demographic, health and social characteristics are all associated with loneliness, with depressive symptoms, living alone, and reduced social participation emerging as the strongest predictors. These findings highlight the need for targeted interventions to reduce loneliness among Chinese older adults. Efforts to address loneliness should prioritize enhancing social support, mental health services, and opportunities for social participation for this vulnerable population.

## Data Availability

The data that support the findings of this study are available from the corresponding author upon reasonable request.

## Abbreviations

2 PL: Two-Parameter Logistic
ADLs: Activities of Daily Living
CES-D: The Center for Epidemiologic Studies Depression Scale
CI: Confidence Intervals
CLASS: China Longitudinal Aging Social Survey
ELSA: English Longitudinal Study of Ageing
GRM: Graded Response Model
HRS: Health and Retirement Study
IRT: Item Response Theory
LSNS-6: Lubben Social Network Scale, a six-item questionnaire
OR: Odd Ratio
UCLA: University of California, Los Angeles
WHO: World Health Organization
